# Evaluation of Serum Glial Cell Line-derived Neurotrophic Factor in Bangladeshi Major Depressive Disorder Patients

**DOI:** 10.7759/cureus.6081

**Published:** 2019-11-06

**Authors:** Rajesh Das, Md Prova Zaman Emon, Sayeeda Fahmee Chowdhury, Sumaiya Huque, Tanzan Zahan, Md Rabiul Islam

**Affiliations:** 1 Pharmacy, University of Asia Pacific, Dhaka, BGD

**Keywords:** major depression, gdnf, serum, bangladesh, neurotrophic factor, mdd

## Abstract

Background

Major depressive disorder (MDD) is a global health burden in the 21^st^ century because of its high rate of prevalence linked with disability, morbidity, and mortality. The actual etiology behind the development of MDD is not understood yet. Various genetic, physiological, biological and environmental factors have been predicted to be involved. As there is currently no sufficient laboratory test for the diagnosis of MDD, it is expected that this investigation can assist in better diagnosis and management of MDD. The present study aimed to evaluate glial cell line-derived neurotrophic factor (GDNF) in MDD patients compared to healthy controls (HCs).

Materials and methods

This case-control study was conducted with 167 participants including 85 MDD patients and 82 age- and sex-matched HCs. A qualified psychiatrist evaluated all the study participants according to the Diagnostic and Statistical Manual of Mental Disorders, 5th edition (DSM-5). The severity of depression was measured by the Hamilton depression rating scale (Ham-D) and the participants with Ham-D score ≥ 7 were considered as cases. Serum GDNF levels were determined by enzyme-linked immunosorbent assay (ELISA) kits (Boster Bio, Pleasanton, CA, USA).

Results

MDD patients and HCs were similar in terms of their socio-demographic profiles. Serum GDNF was found to have no significant alterations in MDD patients when compared to HCs (p > 0.05). Moreover, no significant positive or negative correlation was found between serum levels of GDNF and Ham-D scores in MDD patients.

Conclusions

It can be predicted from the above findings that there is no significant relation between serum GDNF levels and the pathophysiology of depression. This study should be treated as preliminary and further studies with a more homogeneous and larger study population are required to establish these findings.

## Introduction

Depressive disorders are common mental disorders that can occur as early as three years of age and is prevalent across the entire world [1]. Previous global burden of disease (GBD) studies in 1990 noted depressive disorders as a leading cause of burden in comparison to other recognized physical disorders [2]. In GBD 2000, depressive disorders were said to be the third leading cause of burden after lower respiratory infections and diarrheal diseases. It was also mentioned as the leading cause of disability, responsible for 13.4% and 8.3% years of life lived with disability (YLDs) in women and men, respectively [3]. Major depressive disorder (MDD) is a prevalent, heterogeneous illness characterized by depressed mood, anhedonia, and altered cognitive function with an unclear cause. The lifetime prevalence of MDD is approximately 17% of the population which results in tremendous secondary costs to society [4]. Various psychological, biological, genetic, social and environmental factors seemed to affect the prognosis of this depressive disorder as suggested in many recent studies [5]. Many imaging and post-mortem studies in patients with depression or mood disorders have revealed a reduction of particular areas such as the prefrontal cortex, hippocampus, and amygdala in total volume and cell density/size, especially glial cells [6]. The neurotrophic hypothesis explains the pathogenesis of the development of major depression and the effect of antidepressant treatment on depression [7]. Neurotrophic factors are a family of proteins also responsible for the growth, survival, differentiation, and function of developing neurons and the maintenance of mature neurons, and they include brain-derived neurotrophic factor (BDNF) and glial cell line-derived neurotrophic factor (GDNF) [8]. Neurotrophic factors can play crucial roles in the formation and plasticity of neuronal exerts neurotrophic effects on the development and maintenance of glial cells, and can influence the development, survival, and differentiation of dopaminergic, serotonergic, and GABAergic neurons [9].

GDNF, a member of transforming growth factor (TGF)-b superfamily, was originally designated as a trophic factor for midbrain dopamine neurons but later found to have pronounced effects on other neuronal populations [10]. GDNF has been reported to play important roles in higher-ordered brain functions such as cognitive abilities and drug addiction including modulation of synaptic plasticity and the formation of neural circuits as well as, axonal regeneration after injury [11]. A meta-analytic study found that the serum GDNF level is lower in untreated MDD patients than in healthy controls (HCs). However, postmortem and clinical studies have found that the GDNF level is increased in the parietal cortex of recurrent MDD patients and that the plasma GDNF level is increased in elderly MDD patients [12]. GDNF gives different neurotrophic actions by activating the intracellular Ret-tyrosine kinase domain after binding with GDNF receptor-α1 [10,13]. It exerts multiple actions like development, differentiation, maintenance of glial cells [9, 14] and protects both neuron and glial cells from oxidative stress [15]. In the CNS, it also plays a significant role in the protection of catecholaminergic, cholinergic [16], serotonergic and dopaminergic neurons, which regulates noradrenergic and GABAergic pathways [17]. It also has a major role in cognitive functions including learning and memory GDNF receptors present in the hippocampus may be involved in the development of MDD [11].

The etiology of MDD is very complicated and hard to explain. Previously, MDD was diagnosed based on the structured questionnaire of the Diagnostic and Statistical Manual (DSM) and Hamilton rating scale for depression (Ham-D), where often diagnosis and treatment is quite difficult because of a broad range of symptoms. According to the monoamine hypothesis, the inability of neuronal systems to exhibit appropriate adaptive plasticity is associated with MDD and is supported by the alteration of glial cell structure in patients with MDD [18]. The serum level of GDNF was also found debatable in many recent studies [12,19-20]. Hence, we tried to correlate the serum levels of GDNF with the severity of the disease. Some studies in the past indicate higher GDNF levels in MDD patients among study populations whereas others observed vice versa [19-20]. The present study aimed to determine the serum levels of GDNF in MDD patients compared to HCs.

## Materials and methods

Study population

Eighty-seven drug-naive MDD patients with age ranging from 18 to 60 years who met the criteria of DSM, 5^th^ edition (DSM-5) after skillful diagnosis by a qualified psychiatrist were included in this case-control study. All MDD patients who had a history of MDD symptoms, not more than two weeks are recruited from Bangabandhu Sheikh Mujib Medical University (BSMMU), Dhaka, Bangladesh. Eighty-two age- and sex-matched HCs with MDD patients were recruited from different parts of Dhaka city. A structured questionnaire was used in general for the socio-demographic analysis and documentation of the study groups. The Ham-D rating scale was utilized to measure the severity of depression of study populations and patients with scores > 7 were only included in this study. Patients who had a history of other psychiatric disorders like delusional disorders, bipolar disorder, schizophrenia, personality disorder, other co-morbid psychiatric illness, neurological disease or patients with clinical evidence of dementia were excluded from the investigation. Patients with severe or acute medical illnesses, immune disorders, abnormal body mass index (BMI), alcohol or narcotic drug dependency were also left out of this study.

Blood sample collection and storage

From each of the study participants, a 5-ml blood sample was collected from the cephalic vein after overnight fasting in the morning between 8.00 AM to 9.00 AM. All samples are allowed to clot in a fixed place at room temperature for one hour without disturbance. These clotted samples are then centrifuged to extract serum samples at 1000 x g for 15 minutes. The clear serum specimens were then carefully separated and stored at -80° C until further analysis.

Quantification of serum GDNF

Serum GDNF levels were determined by commercially available enzyme-linked immunosorbent assay (ELISA) kits (Boster Bio, Pleasanton, CA, USA). All procedures were completed according to the manufacturer’s instructions. There was no cross-reactivity with other neurotrophic mediators that might be present in the serum. The minimum detectable dose or sensitivity of GDNF was found to be ≤4 pg/mL. The person who carried out the analysis was ignorant about the outcomes.

Statistical analysis

Independent sample t-test and Fisher’s exact test were applied for continuous and categorical variables, respectively. Spearman’s rank correlation test was used to find the relation between serum GDNF levels and the severity of depression. Data were presented as mean ± standard error mean (SEM) and the significance level was set at 5%. All statistical analyses were conducted using Statistical Package for Social Science (SPSS) version 23.0 (IBM Corp., Armonk, NY).

## Results

The socio-demographic profile, biophysical characteristics, clinical outcomes, and laboratory findings of the study population are summarized in Table 1.

**Table 1:** The characteristics, clinical features, and laboratory findings of the study population. Significant p-values ≤ 0.05 at 95% confidence interval; values in bold: p < 0.05 BMI: Body mass index; F/M: Female/male; GDNF: Glial cell line-derived neurotrophic factor; Ham-D: 17-item Hamilton depression rating scale; N: Number; SEM: Standard error mean.

Parameters	Patient group (N = 85)	Healthy controls (N = 82)	
	Mean ± SEM	Mean ± SEM	p-value
Age in years	33.37 ± 1.10	31.13 ± 1.82	0.300
Gender (F/M)	48/37	50/32	0.572
BMI (kg/m^2^)	26.60 ± 0.65	24.83 ± 0.75	0.137
Ham-D score	19.96 ± 0.29	4.96 ± 0.55	<0.001
Serum GDNF (pg/mL)	149.74 ± 6.26	134.58 ± 8.32	0.168

No significant difference was found between the study groups according to age, gender, and BMI. Besides this, we also obtained a significant similarity between study populations in case of education, occupation, economic status, and smoking habit. It was observed that there were no significant alterations of serum levels of GDNF in MDD patients compared with the HCs (p > 0.05).

## Discussion

The result of this case-control study showed no noteworthy change in serum GDNF levels in MDD patients compared to HCs. We found no significant correlations between these neurotrophic factor levels with the severity of depression also. The outcome of our study is supported by many previous studies. Very little work has been done to establish a relationship between GDNF and major depression and among the few findings; most of the results obtained were conflicting [19-20]. In some works, serum GDNF level exhibited no visible alteration between MDD patients and HCs. Whereas in some cases serum GDNF increased considerably in cases of depression while in other investigations it decreased remarkably in patients with MDD. One such study that observed serum GDNF levels did not significantly change in patients with depression was by Rybakowski et al. In their research work, it was found that the serum GDNF levels were not significantly altered in MDD patients compared to HCs [21]. Similarly, Moreira et al. also found no difference in the levels of serum GDNF in their study over three years among a group of 156 women aged between 18 to 60 years suffering from MDD [22]. Although GDNF is a member of the transforming growth factor β family that is widely expressed in the brain, however, there is very little data that states a link between changes in this neurotrophic factor and major depression [23].

Our finding is supported by some previous studies. Cardoso et al. in their research work aimed to evaluate differences among several serum neurotrophic factors such as BDNF, NGF, and GDNF between depressed patients and HCs. The goal was also to verify an association between serum neurotrophic levels and clinical characteristics in a young, depressed population stratified by gender. The outcome of their study demonstrated that although there were significant neurochemical differences in NGF and BDNF which were associated with the clinical features of MDD patients surprisingly there were no remarkable alterations in GDNF that can be related to MDD [24]. Also, no significant difference was found in serum GDNF levels in MDD patients (23.35 ± 5.59 pg/mL) compared to HCs (25.78 ± 6.50 pg/mL) in a study carried out by Lee et al. [25]. One very recent study observed zero difference between serum levels of GDNF in MDD patients with HCs [22]. Interestingly in a study by Naumenko et al. neither GDNF nor BDNF could alter 5‐HTT gene expression, although 5‐HTT is the primary target for selective serotonin reuptake inhibitor (SSRI) antidepressants [26]. Also in another study, GDNF showed no effect on the functional activity of the serotonergic (5HT) system of the brain which is responsible for depression [27, 28]. In a study by Brunoni et al. it was found that plasma GDNF levels in MDD patients did not change significantly after six weeks of antidepressant treatment with sertraline [29]. Results from other studies on GDNF and its relationship to depressive disorder showed no significant differences in GDNF serum level between patients with first depressive episode and HCs. GDNF serum level did not correlate with metabolic parameters except for total cholesterol in depression [30].

According to the present study, no significant alteration in serum GDNF levels was found in MDD patients and although the goal of this study was to identify GDNF as one biomarker of MDD it could not be established. This investigation has some limitations too. We only obtained data from the first depressive phase hence could not associate with the current mood and diagnosis. We also could not obtain any data regarding dietary intake of study populations which might limit the generalization of our findings. The small sample size and not taking account of family history may also interfere with the result of our study.

## Conclusions

To the best of our knowledge, this is the first study regarding the evaluation of serum GDNF levels in MDD patients among the Bangladeshi population. It can be predicted that the finding of unchanged serum GDNF levels indicates a non-significant relation between MDD with these parameters. Despite the exact connection of GDNF in the etiology of depression is not fully understood, still, its neuroprotective capacity might make it a highly interesting future target for antidepressant treatment. As this is only a preliminary study, to understand the exact relation between serum GDNF levels with MDD and to establish these findings as predictors for the assessment of depression risk, further studies with a large and more homogeneous population are required.
